# Crystal structure of (3,5-dimethyl-1*H*-pyrrol-2-yl)di­phenyl­phosphine oxide

**DOI:** 10.1107/S2056989017010994

**Published:** 2017-07-28

**Authors:** Sung Kwon Kang, Eung Man Choi, Kyung-sun Son

**Affiliations:** a Department of Chemistry, Chungnam National University, Daejeon 34134, Republic of Korea

**Keywords:** crystal structure, pyrrole, phosphine oxide, catalysis

## Abstract

The title compound, C_18_H_18_NOP, was obtained during a search for new P,N-containing ligands with the potential to generate precatalysts with chromium(III) for selective ethyl­ene oligomerization. In the crystal, mutual pairs of N—H⋯O=P hydrogen bonds link two mol­ecules into a dimer with individual mol­ecules related by a twofold rotation axis. The P=O bond length of 1.4740 (15) Å is not elongated although the O atom is involved in hydrogen bonding. The crystal structure is further stabilized by van der Waals inter­actions between the dimers, linking the mol­ecules into a three-dimensional network structure.

## Abstract 2

The P=O bond in the crystal structure of (3,5-dimethyl-1*H*-pyrrol-2-yl)di­phenyl­phosphine oxide is not elongated despite its involvement in N—H⋯O=P hydrogen bonding.

## Chemical context   

Mixed bi- and tridentate ligands containing phospho­rus and nitro­gen atoms are highly useful in chromium(III)-catalysed selective ethyl­ene oligomerization (Fliedel *et al.*, 2016 ▸). Several variations of the ligands introduced by chemical modifications can tune the steric and electronic properties of the catalysts, affecting the catalytic behavior in ethyl­ene oligomerization (Agapie, 2011 ▸; McGuinness, 2011 ▸). In search of new P,N-containing ligands, we obtained the title compound from the reaction of 2,4-di­methyl­pyrrole and chloro­diphenyl­phosphine. Herein we present the synthesis and the crystal structure of the title compound, (3,5-dimethyl-1*H*-pyrrol-2-yl)di­phenyl­phosphine oxide, C_18_H_18_NOP, that was obtained by an accidental oxidation reaction.
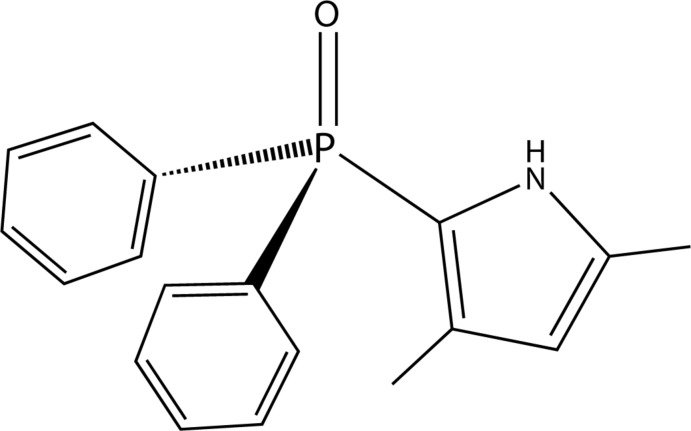


## Structural commentary   

**Figure 1:**
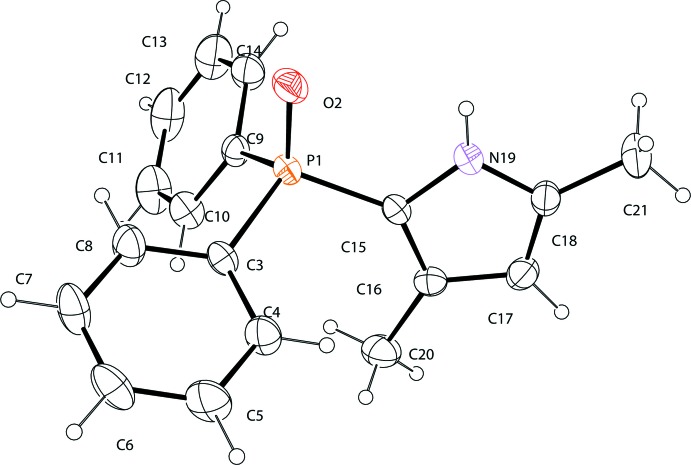
The mol­ecular structure of (I), showing the atom-numbering scheme and displacement ellipsoids at the 30% probability level.

The mol­ecular structure of the title compound, (I), is shown in Fig. 1 ▸. The P=O bond length of 1.4740 (15) Å is virtually identical to that of tri­phenyl­phosphine oxide [1.479 (2) Å; Al-Farhan, 1992 ▸], which is not involved in hydrogen bonding as is the case in the structure of (I). In general, the P=O bond appears to be elongated when involved in hydrogen-bonding inter­actions (Kunz *et al.*, 2011 ▸). In the pyrrole heterocyclic ring of (I), the C15—C16 [1.388 (2) Å] and C17—C18 [1.363 (3) Å] bonds are shorter than the C16—C17 [1.404 (3) Å] bond, even though the pyrrole ring has a delocalized π-system. The bond length of P1—C15 [1.767 (2) Å] to the pyrrole moiety is shorter than those of P1—C3 [1.801 (2) Å] and P1—C9 [1.806 (2) Å] to the C atoms of phenyl rings. Such a slight difference is also observed in the crystal structure of a compound containing the same entity as in (I) (Vélez del Burgo *et al.*, 2016 ▸). The dihedral angle between the O2/P1/C15 plane and the pyrrole ring in (I) is small, 3.89 (5)°.

## Supra­molecular features   

**Figure 2:**
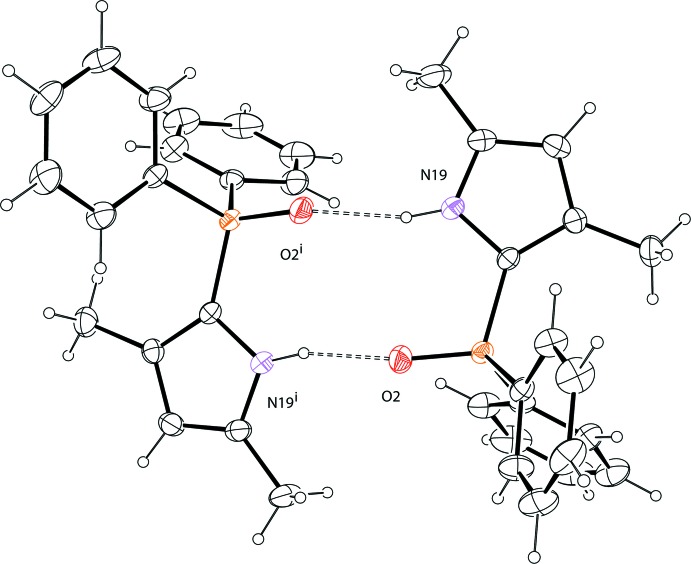
Dimeric structure of (I), showing mol­ecules linked by inter­molecular N19—H19⋯O2^i^ [symmetry code: (i) −*x* + 1, *y*, −*z* + 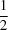] hydrogen bonds (dashed lines).

**Figure 3:**
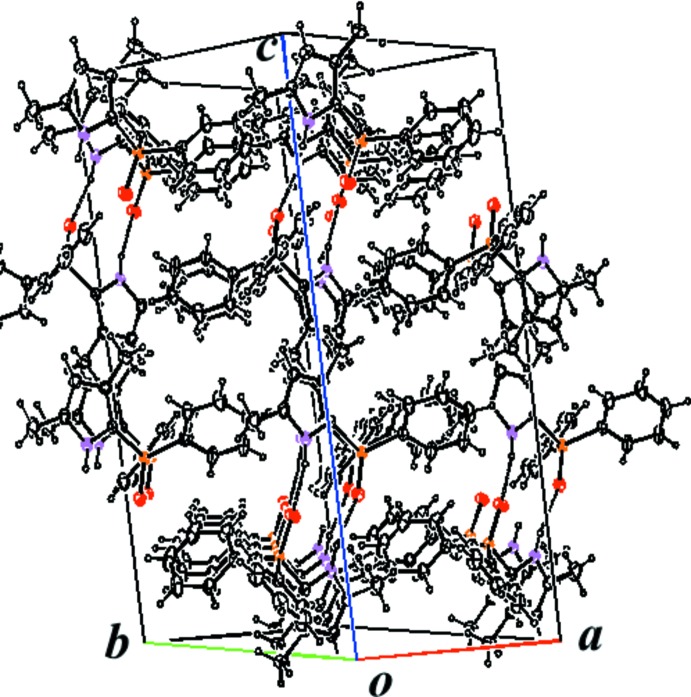
Part of the crystal structure of (I), showing mol­ecules linked by inter­molecular N—H⋯O hydrogen bonds (dashed lines).

Two mutual inter­molecular N19—H19⋯O2^i^ [symmetry code; (i) –*x* + 1, *y*, −*z* + 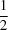] hydrogen bonds between the amino group and the O=P group link two mol­ecules into a dimer (Fig. 2 ▸, Table 1 ▸). The two mol­ecules of the dimer are related by a twofold rotation axis. Apart from van der Waals inter­actions between dimers, there are no other inter­molecular inter­actions that stabilize the three-dimensional crystal packing of (I) (Fig. 3 ▸).

## Database survey   

A search of the Cambridge Structural Database (Version 5.38, update February 2017; Groom *et al.*, 2016 ▸) for compounds containing the (3,5-dimethyl-1*H*-pyrrol-2-yl)di­phenyl­phosphine oxide skeleton revealed only one structure, *viz.* AVPL146MP (Vélez del Burgo *et al.*, 2016 ▸).

## Synthesis and crystallization   

The title compound was prepared by salt elimination after 2,4-di­methyl­pyrrole was treated with tri­methyl­amine and then chloro­diphenyl­phosphine (Moloy & Petersen, 1995 ▸). The ease of *in situ* oxidation of the resulting pyrrole­phosphine derivative led to the formation of the corresponding phosphine oxide ligand (Nyamato *et al.*, 2015 ▸). This new compound was characterized by single crystal X-ray analysis as well as ^1^H, ^13^C, ^31^P NMR, high resolution mass spectrometry, and infrared spectroscopy (see supplementary Figs. S1-S5).

2,4-Di­methyl­pyrrole (0.2 ml, 2 mmol), tri­ethyl­amine (0.34 ml, 3 mmol), and 5 ml of diethyl ether were charged into a Schlenk flask under inert atmosphere. To this solution, chloro­diphenyl­phosphine (0.18 ml, 1 mmol) in 1 ml diethyl ether was added dropwise at 273 K. A colorless precipitate formed immediately. The reaction mixture was then stirred for 10 min at 273 K and heated under reflux for a further 24 h. The precipitate that formed was removed by filtration, and the filtrate was evaporated to dryness under vacuum. The resulting oil was re-dissolved in hexane and filtered. The solvent was removed under vacuum to give the product as a red solid (0.21 g, 0.72 mmol, yield 72%). Single crystals of the title compound were obtained by slow diffusion of hexane into a concentrated solution of the product in tetra­hydro­furan at room temperature. ^1^H NMR (300 MHz, CDCl_3_): δ = 2.17 (*s*, 3H), 2.18 (*s*, 3H), 5.86 (*s*, 1H), 7.27–7.35 (*m*, 11H). ^13^C NMR (150 MHz, CDCl_3_): δ = 12.25 (*d*, *J* = 10.2 Hz), 13.34 (*s*), 110.08 (*d*, *J* = 5.6 Hz), 117.32 (*d*, *J* = 13.5 Hz), 128.40 (*s*), 128.70 (*d*, *J* = 6.5 Hz), 132.39 (*s*), 132.83 (*d*, *J* = 18.5 Hz), 138.03 (*d*, *J* = 8.8 Hz). ^31^P NMR (242 MHz, CDCl_3_): δ = −35.08 (*s*). HRMS (ESI) calculated for C_18_H_19_ONP ([*M* + H]^+^): 296.12043, found: 296.1228. Melting point: 352 K.

## Refinement   

Crystal data, data collection and structure refinement details are summarized in Table 2 ▸. The H atom of the NH group was located in a difference-Fourier map and refined freely. The C-bound H atoms were positioned geometrically and refined using a riding model, with *d*(C—H) = 0.93–0.96 Å, and with *U*_iso_(H) = 1.2*U*_eq_(C) for aromatic-H and 1.5*U*_eq_(C) for methyl-H atoms, respectively.

## Supplementary Material

Crystal structure: contains datablock(s) I. DOI: 10.1107/S2056989017010994/wm5405sup1.cif

Structure factors: contains datablock(s) I. DOI: 10.1107/S2056989017010994/wm5405Isup2.hkl

spectroscopic analysis of the title compound. DOI: 10.1107/S2056989017010994/wm5405sup3.pdf

Supporting information file. DOI: 10.1107/S2056989017010994/wm5405Isup4.cml

CCDC reference: 1564683

Additional supporting information:  crystallographic information; 3D view; checkCIF report

## Figures and Tables

**Table 1:** Hydrogen-bond geometry (Å, °)

*D*—H⋯*A*	*D*—H	H⋯*A*	*D*⋯*A*	*D*—H⋯*A*
N19—H19⋯O2^i^	0.862 (19)	1.92 (2)	2.757 (2)	164.7 (18)

Symmetry code: (i) 
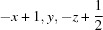
.

**Table 2:** Experimental details

Crystal data	
Chemical formula	C_18_H_18_NOP
*M*_r_	295.30
Crystal system, space group	Monoclinic, *C*2/*c*
Temperature (K)	296
*a*, *b*, *c* (Å)	10.656 (6), 14.765 (8), 20.757 (11)
β (°)	98.378 (8)
*V* (Å^3^)	3231 (3)
*Z*	8
Radiation type	Mo *K*α
μ (mm^−1^)	0.17
Crystal size (mm)	0.29 × 0.27 × 0.25

Data collection	
Diffractometer	Bruker SMART CCD area-detector
Absorption correction	Multi-scan (*SADABS*; Krause *et al.*, 2015 ▸)
*T*_min_, *T*_max_	0.943, 0.967
No. of measured, independent and observed [*I* > 2σ(*I*)] reflections	14781, 3961, 3196
*R*_int_	0.027
(sin θ/λ)_max_ (Å^−1^)	0.670

Refinement	
*R*[*F* ^2^ > 2σ(*F* ^2^)], *wR*(*F* ^2^), *S*	0.046, 0.136, 1.07
No. of reflections	3961
No. of parameters	196
H-atom treatment	H atoms treated by a mixture of independent and constrained refinement
Δρ_max_, Δρ_min_ (e Å^−3^)	0.32, −0.24

Computer programs: *SMART* and *SAINT* (Bruker, 2002 ▸), *SHELXS97* (Sheldrick, 2008 ▸), *SHELXL2013* (Sheldrick, 2015 ▸) and *ORTEP-3 for Windows* and (Farrugia, 2012 ▸).

## supplementary crystallographic information

### Crystal data

**Table:**

C_18_H_18_NOP	*F*(000) = 1248
*M**_r_* = 295.30	*D*_x_ = 1.214 Mg m^−^^3^
Monoclinic, *C*2/*c*	Mo *K*α radiation, λ = 0.71073 Å
*a* = 10.656 (6) Å	Cell parameters from 6973 reflections
*b* = 14.765 (8) Å	θ = 2.4–28.0°
*c* = 20.757 (11) Å	µ = 0.17 mm^−^^1^
β = 98.378 (8)°	*T* = 296 K
*V* = 3231 (3) Å^3^	Block, orange
*Z* = 8	0.29 × 0.27 × 0.25 mm

### Data collection

**Table:**

Bruker SMART CCD area-detector diffractometer	3196 reflections with *I* > 2σ(*I*)
Radiation source: fine-focus sealed tube	*R*_int_ = 0.027
ω scans	θ_max_ = 28.4°, θ_min_ = 2.0°
Absorption correction: multi-scan (*SADABS*; Krause *et al.*, 2015)	*h* = −14→14
*T*_min_ = 0.943, *T*_max_ = 0.967	*k* = −19→19
14781 measured reflections	*l* = −27→27
3961 independent reflections	

### Refinement

**Table:**

Refinement on *F*^2^	0 restraints
Least-squares matrix: full	Hydrogen site location: mixed
*R*[*F*^2^ > 2σ(*F*^2^)] = 0.046	H atoms treated by a mixture of independent and constrained refinement
*wR*(*F*^2^) = 0.136	*w* = 1/[σ^2^(*F*_o_^2^) + (0.069*P*)^2^ + 1.3679*P*] where *P* = (*F*_o_^2^ + 2*F*_c_^2^)/3
*S* = 1.07	(Δ/σ)_max_ = 0.001
3961 reflections	Δρ_max_ = 0.32 e Å^−^^3^
196 parameters	Δρ_min_ = −0.24 e Å^−^^3^

### Special details

**Table:**

Geometry. All esds (except the esd in the dihedral angle between two l.s. planes) are estimated using the full covariance matrix. The cell esds are taken into account individually in the estimation of esds in distances, angles and torsion angles; correlations between esds in cell parameters are only used when they are defined by crystal symmetry. An approximate (isotropic) treatment of cell esds is used for estimating esds involving l.s. planes.

### Fractional atomic coordinates and isotropic or equivalent isotropic displacement parameters (Å^2^)

**Table:**

	*x*	*y*	*z*	*U*_iso_*/*U*_eq_	
P1	0.71878 (4)	0.57612 (3)	0.33110 (2)	0.03972 (14)	
O2	0.65623 (12)	0.55565 (10)	0.26460 (6)	0.0621 (4)	
C3	0.82519 (14)	0.48606 (10)	0.36150 (8)	0.0429 (4)	
C4	0.7970 (2)	0.42449 (12)	0.40734 (11)	0.0602 (5)	
H4	0.7259	0.4330	0.4278	0.072*	
C5	0.8752 (2)	0.34969 (14)	0.42287 (13)	0.0780 (7)	
H5	0.8565	0.3088	0.4542	0.094*	
C6	0.9787 (2)	0.33576 (14)	0.39253 (13)	0.0744 (7)	
H6	1.0294	0.2850	0.4025	0.089*	
C7	1.0074 (2)	0.39616 (16)	0.34775 (14)	0.0796 (7)	
H7	1.0786	0.3871	0.3274	0.096*	
C8	0.93109 (19)	0.47160 (14)	0.33202 (11)	0.0655 (5)	
H8	0.9518	0.5127	0.3013	0.079*	
C9	0.81251 (15)	0.67828 (11)	0.33328 (8)	0.0451 (4)	
C10	0.92531 (18)	0.69087 (13)	0.37448 (11)	0.0597 (5)	
H10	0.9568	0.6453	0.4033	0.072*	
C11	0.9917 (2)	0.77160 (16)	0.37289 (14)	0.0814 (7)	
H11	1.0678	0.7798	0.4005	0.098*	
C12	0.9455 (3)	0.83877 (15)	0.33103 (17)	0.0933 (9)	
H12	0.9906	0.8926	0.3301	0.112*	
C13	0.8345 (3)	0.82778 (15)	0.29083 (15)	0.0909 (9)	
H13	0.8031	0.8744	0.2630	0.109*	
C14	0.7680 (2)	0.74766 (14)	0.29101 (11)	0.0688 (6)	
H14	0.6927	0.7401	0.2626	0.083*	
C15	0.60482 (15)	0.58994 (10)	0.38423 (8)	0.0400 (3)	
C16	0.60900 (17)	0.60509 (11)	0.45053 (8)	0.0468 (4)	
C17	0.48236 (18)	0.60914 (13)	0.46181 (9)	0.0558 (5)	
H17	0.4559	0.6182	0.5021	0.067*	
C18	0.40454 (17)	0.59763 (12)	0.40420 (10)	0.0516 (4)	
N19	0.47866 (13)	0.58591 (9)	0.35719 (8)	0.0435 (3)	
H19	0.4501 (18)	0.5756 (12)	0.3169 (10)	0.049 (5)*	
C20	0.7239 (2)	0.61602 (16)	0.50077 (10)	0.0675 (5)	
H20A	0.7544	0.5574	0.5158	0.101*	
H20B	0.7020	0.6504	0.5368	0.101*	
H20C	0.7888	0.6472	0.4820	0.101*	
C21	0.26291 (19)	0.59778 (19)	0.38760 (13)	0.0812 (7)	
H21A	0.2255	0.6017	0.4269	0.122*	
H21B	0.2357	0.5429	0.3650	0.122*	
H21C	0.2367	0.6489	0.3603	0.122*	

### Atomic displacement parameters (Å^2^)

**Table:**

	*U*^11^	*U*^22^	*U*^33^	*U*^12^	*U*^13^	*U*^23^
P1	0.0339 (2)	0.0405 (2)	0.0439 (3)	0.00439 (15)	0.00249 (17)	−0.00102 (16)
O2	0.0492 (7)	0.0831 (9)	0.0507 (7)	0.0116 (6)	−0.0035 (6)	−0.0094 (6)
C3	0.0362 (8)	0.0360 (7)	0.0547 (9)	0.0020 (6)	0.0006 (7)	−0.0045 (6)
C4	0.0564 (11)	0.0451 (9)	0.0800 (14)	0.0051 (8)	0.0132 (10)	0.0097 (9)
C5	0.0836 (15)	0.0466 (10)	0.1029 (18)	0.0115 (10)	0.0107 (14)	0.0217 (11)
C6	0.0588 (12)	0.0449 (10)	0.1140 (19)	0.0165 (9)	−0.0059 (12)	0.0015 (11)
C7	0.0557 (12)	0.0658 (13)	0.121 (2)	0.0215 (10)	0.0242 (13)	−0.0065 (13)
C8	0.0590 (11)	0.0559 (11)	0.0861 (14)	0.0154 (9)	0.0255 (11)	0.0074 (10)
C9	0.0429 (8)	0.0394 (8)	0.0549 (10)	0.0045 (6)	0.0142 (7)	0.0037 (7)
C10	0.0547 (11)	0.0456 (9)	0.0776 (13)	−0.0055 (8)	0.0049 (10)	−0.0012 (9)
C11	0.0661 (13)	0.0582 (13)	0.122 (2)	−0.0174 (11)	0.0226 (14)	−0.0215 (13)
C12	0.0905 (18)	0.0440 (11)	0.159 (3)	−0.0099 (12)	0.0644 (19)	−0.0032 (14)
C13	0.0995 (19)	0.0523 (12)	0.131 (2)	0.0178 (12)	0.0516 (18)	0.0377 (13)
C14	0.0633 (12)	0.0595 (12)	0.0867 (15)	0.0154 (10)	0.0213 (11)	0.0258 (10)
C15	0.0353 (8)	0.0376 (7)	0.0462 (8)	0.0033 (6)	0.0024 (7)	0.0008 (6)
C16	0.0515 (10)	0.0433 (8)	0.0449 (9)	0.0027 (7)	0.0048 (7)	0.0043 (7)
C17	0.0603 (11)	0.0588 (11)	0.0517 (10)	0.0065 (9)	0.0198 (9)	0.0071 (8)
C18	0.0433 (9)	0.0500 (9)	0.0641 (11)	0.0036 (7)	0.0165 (8)	0.0097 (8)
N19	0.0361 (7)	0.0444 (7)	0.0493 (8)	0.0015 (5)	0.0038 (6)	−0.0002 (6)
C20	0.0699 (13)	0.0776 (14)	0.0510 (11)	0.0019 (11)	−0.0045 (10)	−0.0015 (9)
C21	0.0436 (11)	0.1053 (18)	0.0982 (18)	0.0040 (11)	0.0225 (11)	0.0118 (14)

### Geometric parameters (Å, º)

**Table:**

P1—O2	1.4740 (15)	C12—C13	1.354 (4)
P1—C15	1.7672 (18)	C12—H12	0.9300
P1—C3	1.8011 (17)	C13—C14	1.380 (3)
P1—C9	1.8059 (18)	C13—H13	0.9300
C3—C8	1.377 (3)	C14—H14	0.9300
C3—C4	1.380 (3)	C15—N19	1.381 (2)
C4—C5	1.392 (3)	C15—C16	1.388 (2)
C4—H4	0.9300	C16—C17	1.404 (3)
C5—C6	1.363 (3)	C16—C20	1.497 (3)
C5—H5	0.9300	C17—C18	1.363 (3)
C6—C7	1.355 (4)	C17—H17	0.9300
C6—H6	0.9300	C18—N19	1.353 (2)
C7—C8	1.390 (3)	C18—C21	1.498 (3)
C7—H7	0.9300	N19—H19	0.862 (19)
C8—H8	0.9300	C20—H20A	0.9600
C9—C10	1.383 (3)	C20—H20B	0.9600
C9—C14	1.387 (2)	C20—H20C	0.9600
C10—C11	1.389 (3)	C21—H21A	0.9600
C10—H10	0.9300	C21—H21B	0.9600
C11—C12	1.362 (4)	C21—H21C	0.9600
C11—H11	0.9300		
			
O2—P1—C15	110.49 (9)	C11—C12—H12	119.8
O2—P1—C3	110.63 (8)	C12—C13—C14	120.3 (2)
C15—P1—C3	108.75 (8)	C12—C13—H13	119.9
O2—P1—C9	111.57 (9)	C14—C13—H13	119.9
C15—P1—C9	108.41 (8)	C13—C14—C9	120.4 (2)
C3—P1—C9	106.87 (8)	C13—C14—H14	119.8
C8—C3—C4	118.62 (16)	C9—C14—H14	119.8
C8—C3—P1	118.29 (14)	N19—C15—C16	107.38 (15)
C4—C3—P1	122.60 (14)	N19—C15—P1	117.27 (13)
C3—C4—C5	120.0 (2)	C16—C15—P1	135.35 (13)
C3—C4—H4	120.0	C15—C16—C17	106.22 (15)
C5—C4—H4	120.0	C15—C16—C20	127.80 (17)
C6—C5—C4	120.6 (2)	C17—C16—C20	125.97 (17)
C6—C5—H5	119.7	C18—C17—C16	108.97 (16)
C4—C5—H5	119.7	C18—C17—H17	125.5
C7—C6—C5	119.78 (18)	C16—C17—H17	125.5
C7—C6—H6	120.1	N19—C18—C17	107.71 (16)
C5—C6—H6	120.1	N19—C18—C21	120.57 (19)
C6—C7—C8	120.4 (2)	C17—C18—C21	131.71 (19)
C6—C7—H7	119.8	C18—N19—C15	109.72 (16)
C8—C7—H7	119.8	C18—N19—H19	124.2 (13)
C3—C8—C7	120.6 (2)	C15—N19—H19	126.0 (13)
C3—C8—H8	119.7	C16—C20—H20A	109.5
C7—C8—H8	119.7	C16—C20—H20B	109.5
C10—C9—C14	118.60 (18)	H20A—C20—H20B	109.5
C10—C9—P1	123.76 (13)	C16—C20—H20C	109.5
C14—C9—P1	117.65 (15)	H20A—C20—H20C	109.5
C9—C10—C11	120.0 (2)	H20B—C20—H20C	109.5
C9—C10—H10	120.0	C18—C21—H21A	109.5
C11—C10—H10	120.0	C18—C21—H21B	109.5
C12—C11—C10	120.2 (2)	H21A—C21—H21B	109.5
C12—C11—H11	119.9	C18—C21—H21C	109.5
C10—C11—H11	119.9	H21A—C21—H21C	109.5
C13—C12—C11	120.5 (2)	H21B—C21—H21C	109.5
C13—C12—H12	119.8		
			
O2—P1—C3—C8	66.62 (17)	C10—C11—C12—C13	0.3 (4)
C15—P1—C3—C8	−171.86 (15)	C11—C12—C13—C14	−1.1 (4)
C9—P1—C3—C8	−55.02 (17)	C12—C13—C14—C9	1.4 (4)
O2—P1—C3—C4	−105.19 (17)	C10—C9—C14—C13	−0.7 (3)
C15—P1—C3—C4	16.33 (17)	P1—C9—C14—C13	179.28 (17)
C9—P1—C3—C4	133.17 (16)	O2—P1—C15—N19	−3.94 (14)
C8—C3—C4—C5	0.0 (3)	C3—P1—C15—N19	−125.55 (12)
P1—C3—C4—C5	171.80 (16)	C9—P1—C15—N19	118.61 (12)
C3—C4—C5—C6	−0.9 (3)	O2—P1—C15—C16	176.30 (16)
C4—C5—C6—C7	1.3 (4)	C3—P1—C15—C16	54.69 (18)
C5—C6—C7—C8	−0.8 (4)	C9—P1—C15—C16	−61.15 (18)
C4—C3—C8—C7	0.5 (3)	N19—C15—C16—C17	0.45 (18)
P1—C3—C8—C7	−171.64 (18)	P1—C15—C16—C17	−179.78 (14)
C6—C7—C8—C3	−0.1 (4)	N19—C15—C16—C20	−179.03 (18)
O2—P1—C9—C10	−145.38 (16)	P1—C15—C16—C20	0.7 (3)
C15—P1—C9—C10	92.73 (17)	C15—C16—C17—C18	−0.5 (2)
C3—P1—C9—C10	−24.33 (18)	C20—C16—C17—C18	178.98 (18)
O2—P1—C9—C14	34.61 (17)	C16—C17—C18—N19	0.4 (2)
C15—P1—C9—C14	−87.28 (16)	C16—C17—C18—C21	−178.5 (2)
C3—P1—C9—C14	155.66 (14)	C17—C18—N19—C15	−0.10 (19)
C14—C9—C10—C11	−0.1 (3)	C21—C18—N19—C15	178.96 (18)
P1—C9—C10—C11	179.85 (16)	C16—C15—N19—C18	−0.23 (18)
C9—C10—C11—C12	0.4 (3)	P1—C15—N19—C18	179.95 (11)

### Hydrogen-bond geometry (Å, º)

**Table:**

*D*—H···*A*	*D*—H	H···*A*	*D*···*A*	*D*—H···*A*
N19—H19···O2^i^	0.862 (19)	1.92 (2)	2.757 (2)	164.7 (18)

Symmetry code: (i) −*x*+1, *y*, −*z*+1/2.
