# Supplementary material for: Crystal structure of (3,5-dimethyl-1H-pyrrol-2-yl)di­phenyl­phosphine oxide
Source: Acta Crystallogr E Crystallogr Commun. 2017 Jul 28;73(Pt 8):1268–70. doi: 10.1107/S2056989017010994 (PMC5598863; doi:10.1107/S2056989017010994)
Supplement: Supplementary file 3 [file e-73-01268-sup3.pdf]

Crystal structure of (3,5-dimethyl-1H-pyrrol-2-yl)diphenylphosphine oxide

Sung Kwon Kang, Eung Man Choi, and Kyung-sun Son*

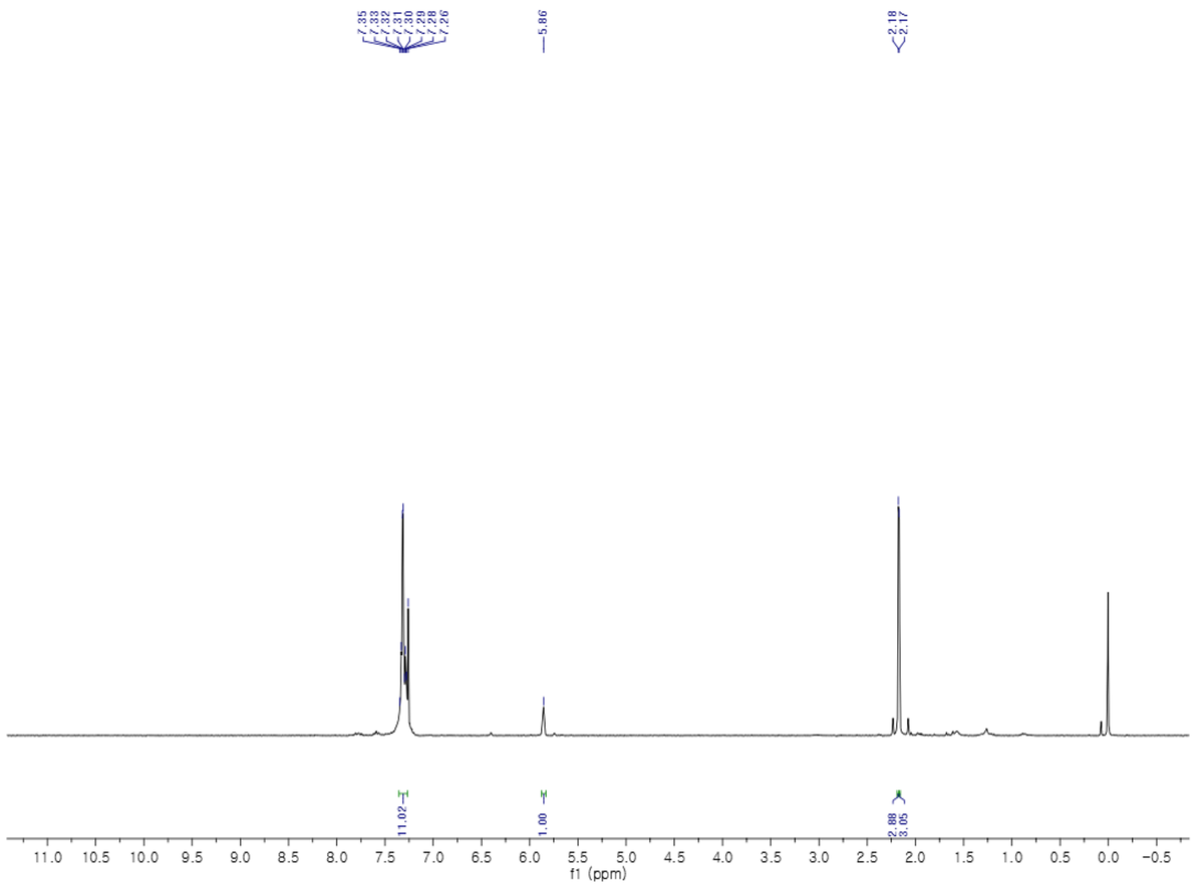

Fig. S1. ^{1}H NMR of the title compound (in $CDCl_3$)

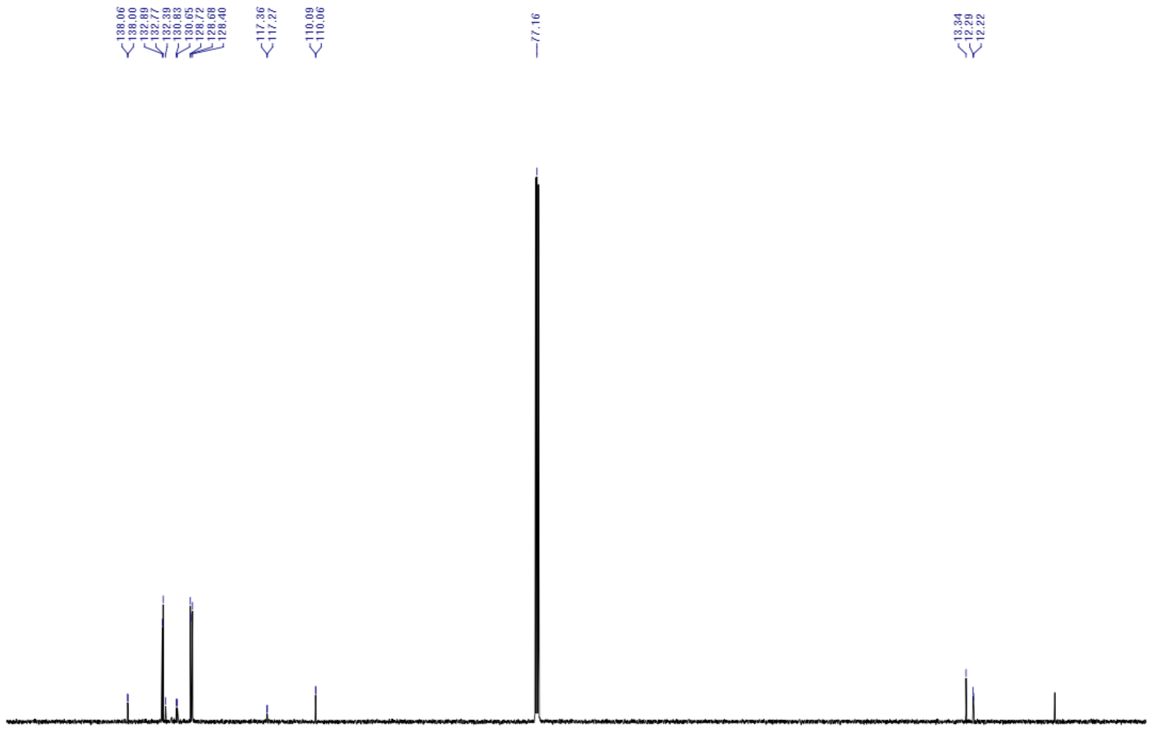

Fig. S2. ^{13}C NMR of the title compound (in $CDCl_3$)

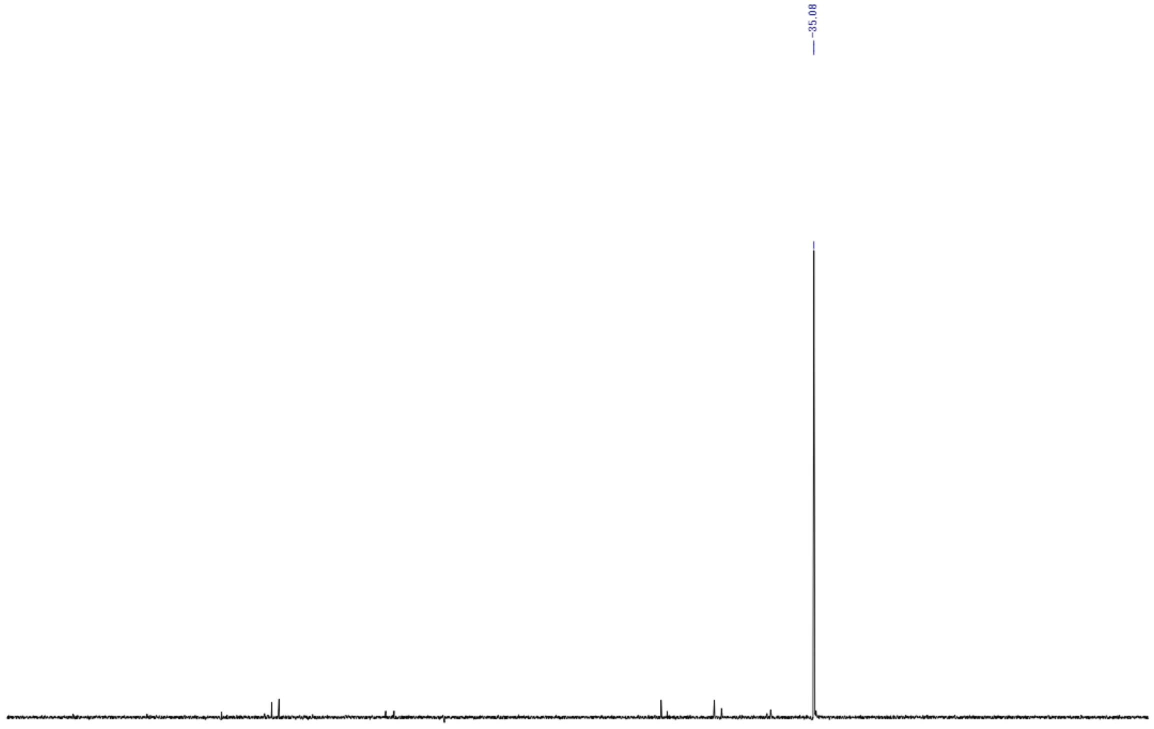

Fig. S3. ^{31}P NMR of the title compound (in $CDCl_3$)

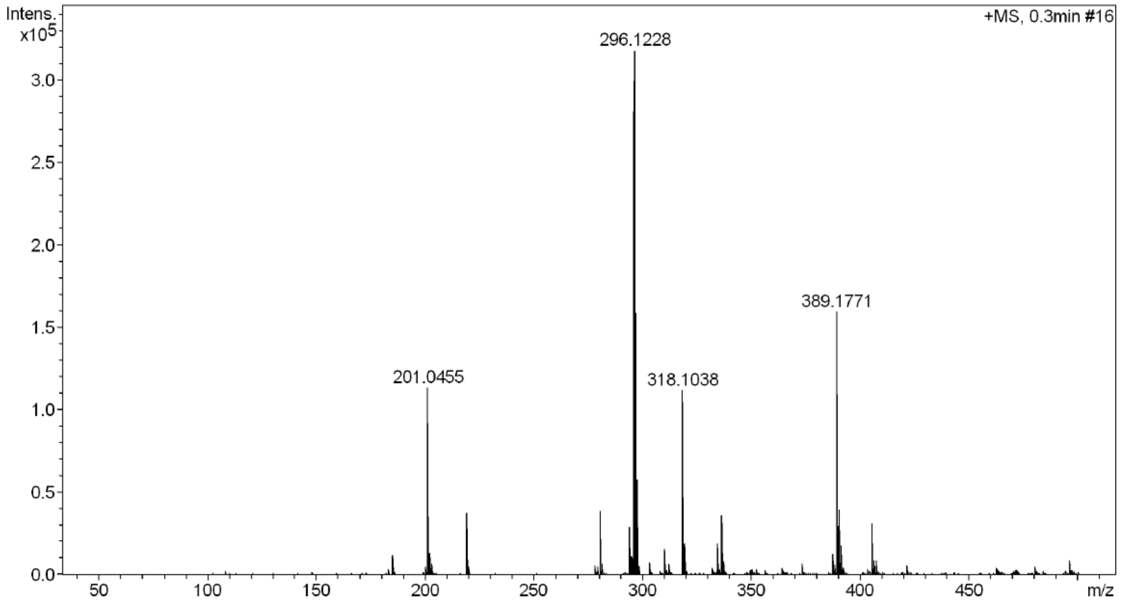

Fig. S4. High-resolution mass spectrum of the title compound

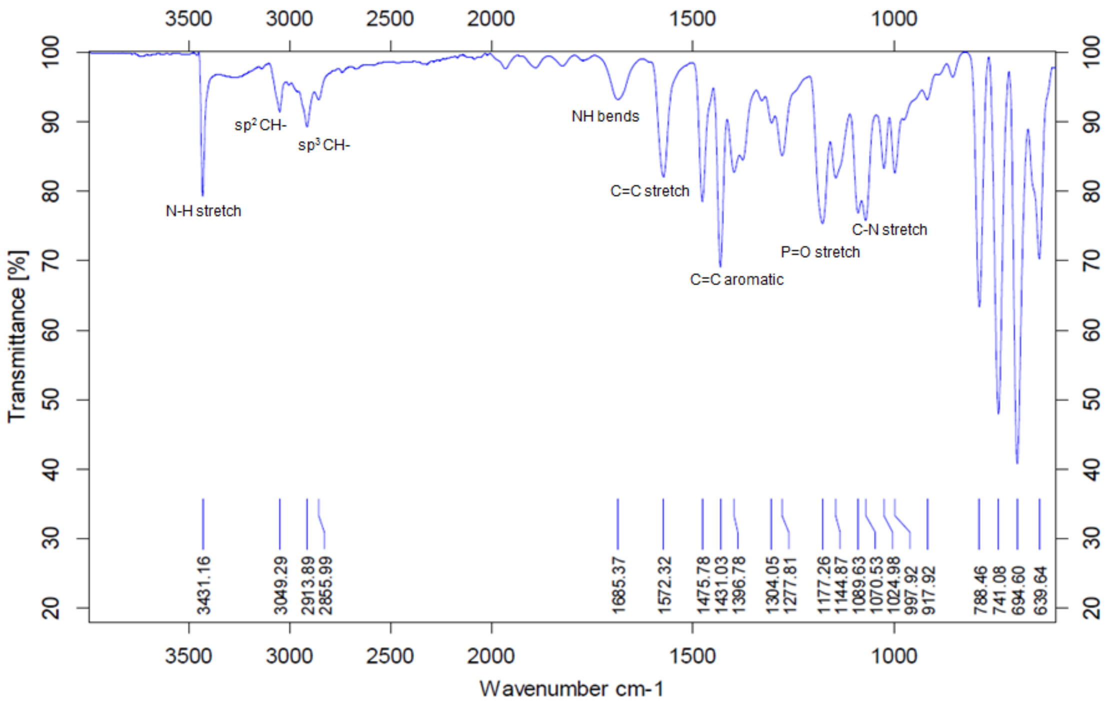

Fig. S5. Infrared spectrum of the title compound
